# Genetic Risk Factors: Their Function and Comorbidities in Alzheimer's Disease

**DOI:** 10.4061/2011/925362

**Published:** 2011-08-11

**Authors:** Mikko Hiltunen, Lars Bertram, Aleister J. Saunders

**Affiliations:** ^1^Institute of Clinical Medicine-Neurology, University of Eastern Finland, 70211 Kuopio, Finland; ^2^Neuropsychiatric Genetics Group, Max Planck Institute for Molecular Genetics, 14195 Berlin, Germany; ^3^Department of Biology, Drexel University, Philadelphia, PA 19104, USA

Alzheimer's disease (AD) is an epidemiologically complex disorder, in which both genetic and environmental factors play important roles contributing to disease susceptibility. Identification of these risk factors is crucial as they may provide new avenues for the identification of novel disease biomarkers as well as for the design of intervention approaches. Several novel AD susceptibility genes with small risk effects have been recently identified by employing genetic association analyses, in particular by those using a genome-wide approach. For most of the newly identified AD risk factors, however, the biological mechanisms driving these associations remain elusive, emphasizing the need for comprehensive functional characterization of these genes and for determining their relevance for AD pathogenesis. In addition, epidemiological and clinical studies have revealed that certain comorbidities often precede or cooccur with AD. These are often correlated with modifiable life-style factors potentially providing promising alternative routes to be exploited in treatment studies. For this special issue of the *International Journal of Alzheimer's Disease*, we invited investigators to contribute original research and review articles that stimulate efforts to identify novel molecular targets involved in AD pathogenesis. Eventually, we selected to include eight articles on the topic, which we believe to be of particular interest to the readers of the journal.

The first set of four studies in this special issue elucidates a number of different genetic aspects of AD. The study by G. Hamilton et al. assessed the impact of recently identified AD genome-wide association signals on cognitive functioning in two birth cohorts from Scotland. Their strongest results implicate a haplotype at the *TRAPPC6A* locus in individuals lacking the *APOEε*4 allele. Less-pronounced effects on cognition are also observed for genetic variants in *APP* and *BIN1*. The study by L. Polito et al. investigated the potential role of *SLC6A4*, a serotonin transporter highly expressed in the brain, in contributing to AD risk. While they found nominally significant risk effects in their own case-control sample from Italy, combining these data with those from other groups yields a more ambiguous answer. The study by R. Dominici et al. followed a similar approach investigating the potential effects of DNA sequence variants in G protein-coupled receptor 3 (GPR3) in AD cases and controls from Italy. In agreement with recent genome-wide association studies, they found no evidence that *GPR3* is involved in AD epidemiology. Finally,the paper by E. Blom et al. chose a more functionally orientated approach to AD genetics. The authors performed a genome-wide gene expression study in transgenic mouse models of AD, which suggested differences in expression patterns in genes of the Wnt pathway. Validation experiments in human brain samples confirm these findings and suggest that *TCF7L2* and *MYC* show the largest expression differences in AD versus control subjects.

The second set of studies focus on comorbidities and life-style factors that are associated with AD. In the paper by V. Leinonen et al., investigators assessed the well-known AD-related biomarkers, such as A*β*42, tau protein, and inflammatory components from the cerebrospinal fluid samples obtained from the patients of idiopathic normal pressure hydrocephalus (iNPH). As AD is the most important differential diagnosis for iNPH, brain biopsy samples obtained from NPH patients provide valuable information on pathogenic events taking place in early phase of AD. The paper by G. Pasinetti et al. summarizes the effects of caloric intake, dietary life-style and macronutrient composition on the risk of AD. More specifically, the investigators discuss how certain cardiovascular and diabetic conditions can induce an increased susceptibility for AD and provide potentialmechanisms through which this may occur. The paper by E. Tuminello et al. provides a comprehensive review related to the hypothesis of apolipoprotein E (protein: apoE; gene: *APOE*) antagonistic pleiotropy. The leading hypothesis is that the *APOEε*4 allele may be beneficial in earlier ages, while it leads to cognitive decline later in life. Finally, the last paper by V. Leduc et al. highlights the pleiotropic roles and the structure-function relationship of apoE particularly in the lipid homeostasis-related events in AD and cardiovascular diseases.

Taken together, we believe that the articles included in this special issue of the *International Journal of Alzheimer's Disease* shed new light on a number of different aspects of AD pathogenesis. In concert with the work from hundreds of other AD laboratories worldwide, these exciting new data will hopefully translate into the identification of novel biomarkers and the development of new therapeutic strategies to efficiently diagnose and treat this devastating disorder.

*Mikko Hiltunen* *Mikko Hiltunen* *Lars Bertram* *Lars Bertram* *Aleister J. Saunders* *Aleister J. Saunders*

